# Targeting biofilm-driven antibiotic resistance: emerging mechanisms and next-generation therapeutic interventions

**DOI:** 10.3389/fmicb.2026.1823476

**Published:** 2026-04-23

**Authors:** Abdulkarim Mbaraka, Ritu Raj Meena, Ekta Menghani, Nidhi Verma

**Affiliations:** 1 Department of Biochemistry and Molecular Biology, Muhimbili University of Health and Allied Sciences (MUHAS), Dar es Salaam, Tanzania; 2 Department of Biotechnology, JECRC University, Jaipur, Rajasthan, India; 3 Mahatma Gandhi Institute of Health Informatics (MGIHI), Mahatma Gandhi University of Medical Sciences and Technology, Jaipur, Rajasthan, India

**Keywords:** antimicrobial resistance, biofilm-associated antibiotic resistance, combination therapy, ESKAPE pathogens, extracellular matrix

## Abstract

Biofilm mediated antimicrobial resistance (AMR) has become a critical global health and economic challenge, affecting both community and healthcare settings. Microbial Biofilms significantly enhance the antibiotic tolerance and cause the persistent and device-associated infections via limited drug penetration, degradation of antibiotics, and assist horizontal gene transfer. Biofilm-mediated antimicrobial resistance remains a major obstacle to treating infectious diseases today. Biofilms can boost antibiotic tolerance by up to 1,000 times and lead to chronic, persistent, and device-associated infections. The lack of FDA-approved anti-biofilm drugs highlights the urgent need for new therapeutic strategies and mechanistic insights. Redefining the treatment landscape and improving outcomes for resistant infections could be achieved through a multi-platform therapeutic approach. This review summarizes recent developments in our knowledge of how biofilms contribute to antibiotic resistance and highlights new therapeutic strategies, such as nanotechnology, antimicrobial peptides, bacteriophage-derived enzymes, quorum-sensing inhibitors, CRISPR-based tools, microbiome engineering, and AI-driven drug discovery.

## 1 Introduction

Antimicrobial resistance (AMR) is recognized as one of the most significant global health threats, causing an estimated 700,000 deaths annually due to drug-resistant infections, with projections rising to 10 million by 2050 if immediate interventions are not implemented. The emergence of multidrug-resistant bacteria has diminished the effectiveness of standard antibiotics, leading to prolonged hospital stays, increased healthcare costs, and elevated mortality rates. AMR arises from complex mechanisms, including horizontal gene transfer, genetic mutations, and selective pressures resulting from the misuse and overuse of antibiotics (Nazir et al., 2025). The failure with AMR linked treatment strategies have significantly affected by microbial biofilm formation an underappreciated contributor. These biofilms are prearranged microbial communities sheathed within a self-produced extracellular polymeric substance (EPS) matrix that adheres to biotic or abiotic surfaces. This matrix acts as a physical and chemical barrier due to limiting antimicrobial dissemination, sequestering antibiotics, and varying limited microenvironments (Agarwal et al., 2025). Within biofilms, bacterial communities’ show physiological heterogeneity, including slow-growing and metabolically inactive subpopulations that exhibit reduced antibiotics susceptibility. In addition, the close spatial association of cells within biofilms facilitate horizontal gene transfer, thereby accelerating the dissemination of resistance determinants (Liu et al., 2024). The clinically revalent ESKAPE microorganisms that form biofilms are *Enterococcus faecium, Staphylococcus aureus, Klebsiella pneumonia, Acinetobacter baumannii, Pseudomonas aeruginosa*, and *Enterobacter species* (Abebe et al., 2026b; Sharma et al., 2023).

The ESKAPE pathogens are of special concern when talking about AMR as they can develop biofilms and resist antibacterial therapy (Sarkar et al., 2024). A number of these organisms enthusiastically colonize in medical devices such as urinary catheters, ventilators, prosthetic joints, and cardiac implants, as well as host tissues including chronic wounds and the lungs of patients with cystic fibrosis (Mendhe et al., 2023). Biofilm formation in healthcare settings promotes persistent infection and noticeably decreases the antimicrobial efficacy, portrait suppression difficult even when pathogens appear susceptible under planktonic testing conditions.

While biofilm formation can confer ecological advantages to microbial communities, including enhanced survival under environmental stress, its consequences in clinical contexts are overwhelmingly detrimental. Biofilm-associated bacteria exhibit increased tolerance not only to antibiotics but also to disinfectants and host immune responses, contributing to infection chronicity and recurrence (Muhammad et al., 2020). Consequently, biofilm production by clinically important pathogens such as *S. aureus*, *K. pneumoniae*, and *A. baumannii* has been strongly associated with poor clinical outcomes and long-term disease persistence (Shineh et al., 2023). Importantly, antimicrobial resistance and antimicrobial tolerance represent distinct but interconnected phenomena in biofilm-associated infections. Resistance refers to the heritable ability of microorganisms to grow in the presence of antimicrobial concentrations that would otherwise inhibit or kill susceptible strains, typically mediated by stable genetic changes. In contrast, antimicrobial tolerance is a reversible, non-heritable state in which bacterial cells survive transient exposure to high antibiotic concentrations without an increase in minimum inhibitory concentration. Biofilms predominantly promote tolerance rather than classical resistance by inducing metabolic dormancy, stress responses, and the formation of persister cells. This distinction is clinically critical, as tolerance-mediated survival within biofilms provides a persistent reservoir of viable bacteria that sustains chronic infection and creates conditions that favor the subsequent evolution of true genetic resistance under prolonged antimicrobial pressure.

## 2 Mechanistic insights into biofilm-induced multidrug resistance

**Figure 1:**
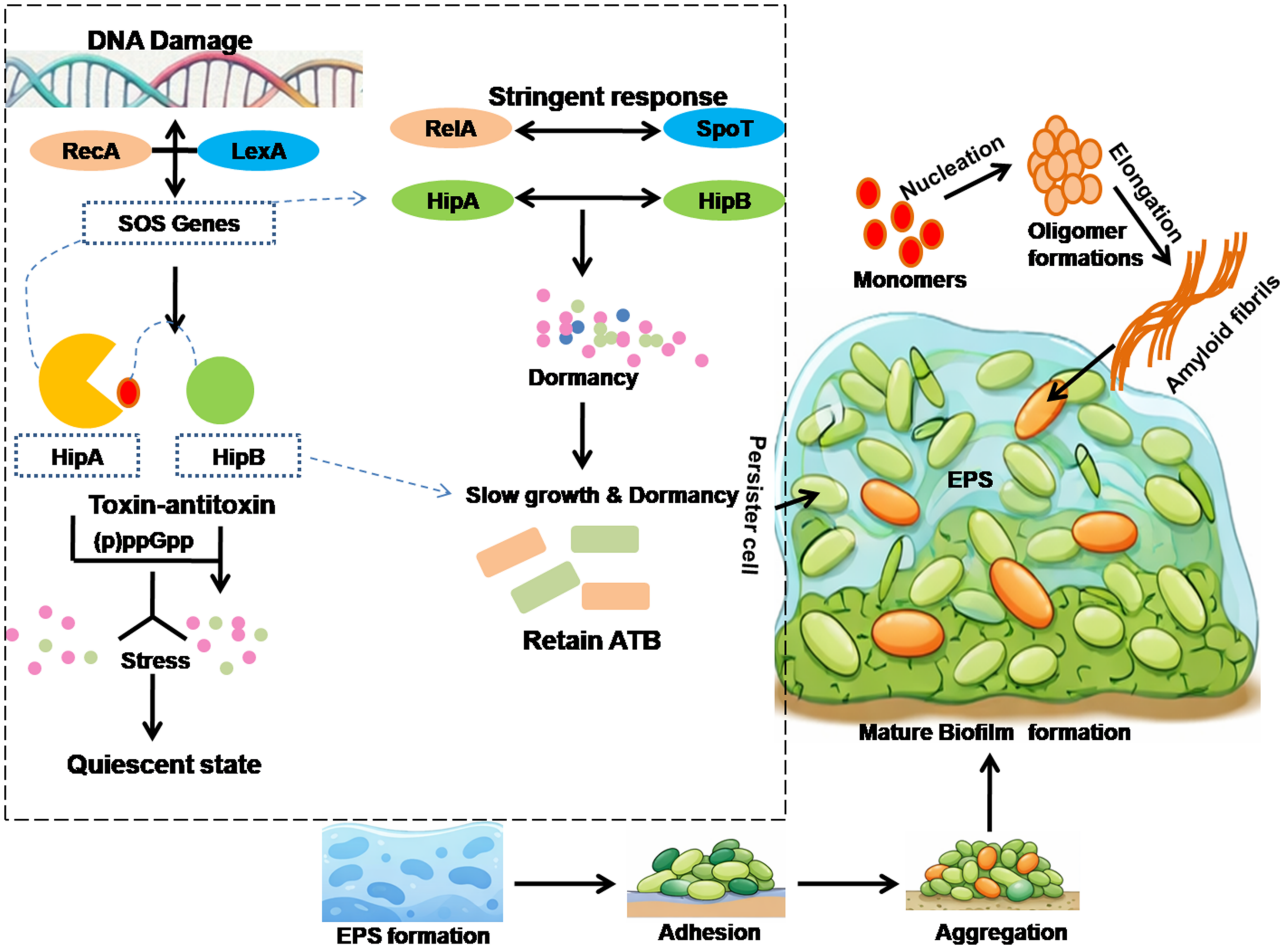
Schematic illustration of persister cell formation within biofilms. The molecular mechanism of persister cell formation in biofilms, mediated by the SOS response, toxin-antitoxin system, and stringent response, promotes antibiotic tolerance.

Microorganisms within biofilms are protected by a matrix of EPS, which acts as a barrier against competitors, disinfectants, antibiotics, and other sterilizing agents (Molina-Santiago et al., 2019). This matrix consists of self-produced extracellular DNA (eDNA), polysaccharides, extracellular proteins, and amyloids, providing resistance through enzymatic degradation and electrostatic interactions as shown in Figure 1. In hospital-acquired and chronic infections, Neutrophil Extracellular Traps (NETs) and eDNA from necrotic neutrophils combine with bacterial eDNA to form a protective layer that shields against antibiotics such as tobramycin and β-lactams and prevents immune clearance. This phenomenon, known as the inoculum effect, reduces antibiotic efficacy (Baz et al., 2024). Recent advances in structural biology, including cryo-electron microscopy and atomic force microscopy, have revealed more about the organization of biofilm EPS. eDNA acts as a structural scaffold, facilitates horizontal gene transfer, and sequesters cations, enhancing antimicrobial tolerance (Goodman and Bakaletz, 2022). eDNA plays a crucial role in initial bacterial adhesion and promotes intercellular aggregation, microcolonies formation, and the development of mature biofilm architecture. The filamentous networks formed by eDNA enhance the mechanical stability and integrity of the biofilm matrix. Additionally, eDNA can modulate immune responses, participate in environmental acidification, serve as a nutrient source during starvation, and facilitate biofilm dispersion and electron transfer processes (Campoccia et al., 2021). Despite of this, biofilm formation is highly synchronized by interrelated signaling pathways such as Cyclic di-guanosine monophosphate (c-di-GMP) act as second messenger that require for transition from planktonic to sessile form. The cell communication in microbial communities further trigged by Quorum sensing (QS) mechanism via population density-dependent signaling, involved in EPS synthesis, virulence, and matrix formation. These regulatory systems cooperatively direct the biofilm formation, thereby enhancing persistence and resistance to antimicrobial therapies (Abebe et al., 2026a).

Due to its central role in biofilm integrity and pathogenicity, eDNA is considered a promising therapeutic target for the prevention and treatment of chronic biofilm-associated infections. Bacterial amyloid fibers such as TasA-TapA, curli, and BAP provide mechanical stability and adhesion, while outer membrane vesicles deliver enzymes, toxins, and resistance determinants within the biofilm matrix (Sleutel et al., 2023). These components create a dynamic, protective microenvironment that impedes antibiotic penetration and promotes persistence. Persister cells in deeper biofilm layers serve as reservoirs for AMR genes, resisting bactericidal drugs by slowing growth and macromolecular synthesis. Their formation is regulated by stress-response networks, including the SOS response mediated by RecA–LexA, toxin–antitoxin systems such as HipA–HipB, and the stringent response governed by RelA/SpoT-dependent (p)ppGpp accumulation shown in Figure 1. These pathways induce metabolic dormancy, reduce the activity of antibiotic targets, and enhance survival under hostile conditions (Podlesek and Žgur Bertok, 2020).

Persister cells can repopulate biofilms after treatment ends, contributing to chronic and relapsing infections. Some intrinsic factors include efflux pump activation, reduced membrane permeability from porin loss, enzymatic drug inactivation, and changes to antibiotic targets are also responsible for the emergence of AMR (Belay et al., 2024). The outer membrane vesicles (OMVs) and eDNA in the matrix facilitate horizontal gene transfer (HGT) and antibiotic resistance through mechanisms like transduction, conjugation, and OMV-mediated gene transfer, particularly among Gram-negative pathogens (Dell’Annunziata et al., 2021). These reduce how well antibiotics work in the cell due to bacterial biofilm formation. These processes facilitate the dissemination of antimicrobial resistance genes within bacterial populations and contribute to the persistence of AMR.

In polymicrobial biofilms, QS regulates cell signaling, affecting virulence, antibiotic susceptibility, gene transfer, biofilm formation, virulence factor production, and antimicrobial tolerance (Liu et al., 2024). Additionally, Host-specific factors such as tissue type, immune status, and local environmental conditions strongly influence biofilm formation and structure. *In vivo*, biofilms exhibit greater heterogeneity than *in vitro* models, characterized by spatial differences in oxygen levels, nutrient availability, pH, and microbial composition (Obando and Serra, 2024). For example, pulmonary biofilms develop in hypoxic, mucus-rich environments, while chronic wound biofilms are shaped by nutrient-rich and inflammatory conditions (Sauer et al., 2022). This variability affects antimicrobial penetration, bacterial metabolism, and treatment outcomes. Additionally, biofilms evade immune responses through mechanisms such as inhibiting phagocytosis, altering inflammatory signaling, and increasing resistance to oxidative stress (Grari et al., 2025). A thorough understanding of these host–pathogen interactions is essential for developing effective, targeted anti-biofilm therapies.

Although recent studies have identified biofilm-disrupting molecules from bacteria, fungi, plants, and marine organisms, none have received approval from regulatory agencies such as the FDA or India’s Central Drug Control Organization (Qiu et al., 2025). Most anti-biofilm compounds have been found through random screening or natural product isolation, and their mechanisms are often unclear. Many are only effective at high micromolar concentrations, highlighting the need for further optimization. Understanding the mechanisms of current biofilm disruptors may lead to more effective treatments. New agents, including imidazole, phenol, indole, and furanone derivatives, have been synthesized, but increasing antibiotic resistance underscores the need for improved therapies (Qin et al., 2026). Developing small molecules that inhibit biofilm formation or enhance existing antibiotics is promising. Drug repurposing also offers potential, but further research is required to identify new and effective therapies (Verma et al., 2025). Continued research and development of targeted biofilm-disrupting agents are critical to combating biofilm infections and improving patient outcomes.

## 3 Therapeutic strategies related to combat multidrug resistance associated with biofilm formations

**Figure 2:**
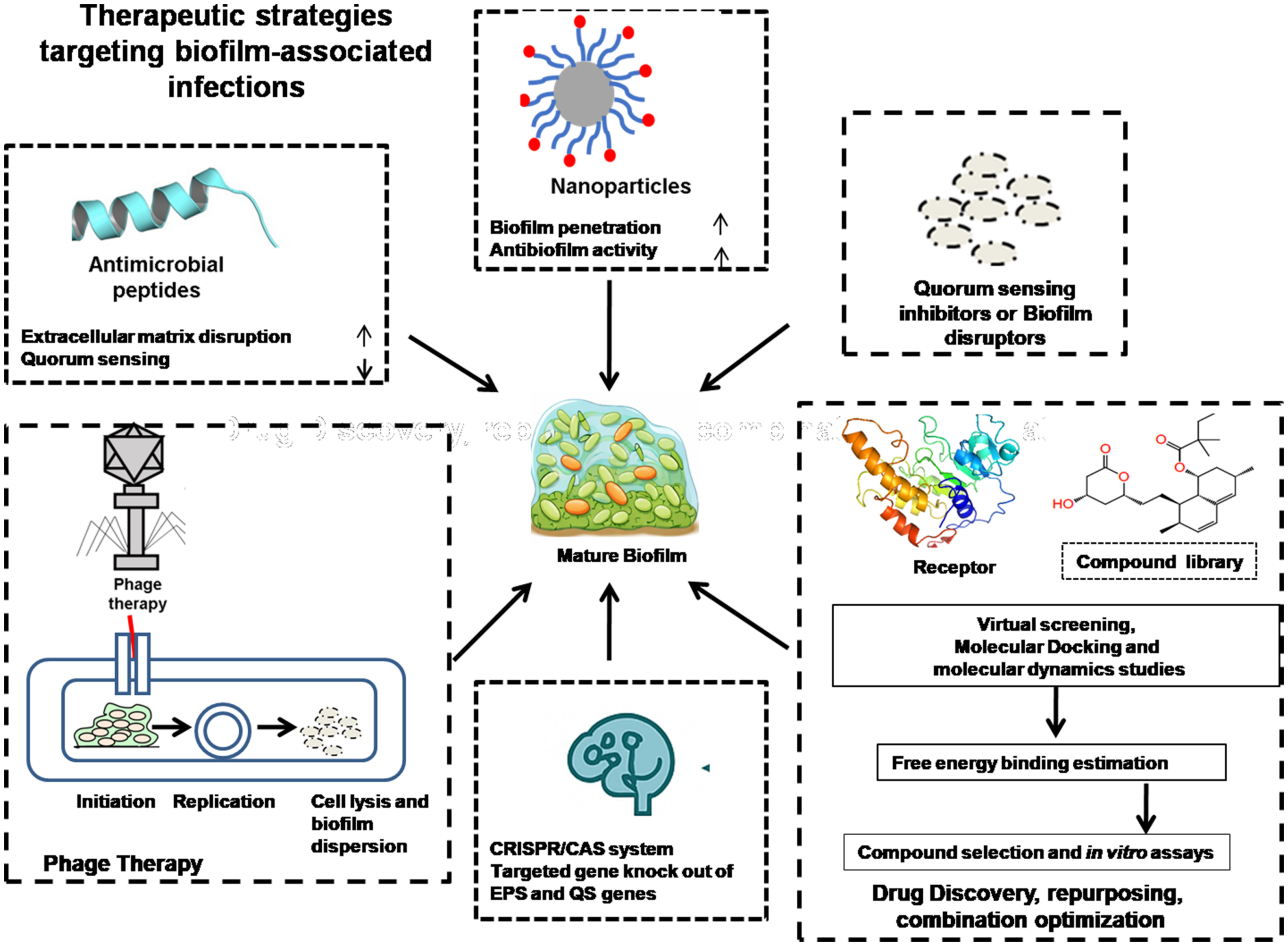
Represents the therapeutic strategies to overcome antimicrobial resistance by targeting biofilm formation.

To overcome the multidrug resistance (MDR) due to microbial biofilms remains a significant health concern, and it accounts for 1.27 million deaths across the world. A considerable percentage of healthcare-related infections are now antibiotic-resistant. In its 2019 report, the CDC pointed out AMR as a major threat to human health, as well as to medicine, animals, and farming. Antibiotic overuse and misuse have resulted in the emergence of resistant microbial strains, making antimicrobial treatment less effective (Bhardwaj et al., 2025). There has been a reaction to this emerging problem through the development of a number of different therapeutic approaches that provide new means of fighting MDR and biofilm infections (Abebe and Birhanu, 2023) shown in Figure 2.

### 3.1 Nanotechnology-based approaches

Nanotechnology has been recently incorporated with multiple interdisciplinary approaches to tackle AMR and biofilm formation. Strategies like nanoparticle-based drug delivery, nanobiotics, and antibacterial vaccine development are on the rise to manage MDR strains (Muteeb, 2023). Nanoparticles (NPs) function as effective transporters of antibiotics and natural antimicrobial compounds in boosting their delivery in biofilms to target effectively resistant strains. Gold, silver, copper, and zinc oxide nanoparticles have been found to fight MRSA and ESCAPE pathogens as excellent therapeutic drugs. Also, nanocarriers like liposomes, polymeric nanoparticles, and dendrimers enhance bioavailability, diminish toxicity, and avoid drug buildup. Nanotechnology-based photothermal therapy with functional antibodies is another promising approach for biofilm destruction. Due to their minute dimensions and immense surface area, nanoparticles can permeate biofilm matrices and selectively address microbial species, providing a powerful tool in combating biofilm-mediated infections.

NPs are available in various formulations, such as organic NPs like liposomes, which have outer lipid layers that bear hydrophilic or hydrophobic drugs. Organic NPs are biodegradable and non-toxic and hence are potential candidates for therapeutic applications. Aerosolized antimicrobial agents like ciprofloxacin, tobramycin, and amikacin have been effectively employed in the treatment of acute and chronic respiratory infections. Besides organic NPs, inorganic nanoparticles such as metallic NPs have also exhibited potent antibacterial activity and are being investigated for combinatorial therapy with conventional antibiotics (Muhammad et al., 2020). Organic and inorganic NPs have been used to cure a variety of pathogenic diseases.

Hybrid NPs, which consist of both organic and inorganic components, are being considered due to their improved drug delivery, enhancing biological activity, treatment efficiency, and minimizing toxicity. The mechanisms through which NPs inhibit microbes are multifaceted and varied, including processes such as the production of reactive oxygen species, disruption of membranes, and interactions with intracellular structures (Gupta et al., 2019). Nevertheless, though full of promise, NPs are also environmentally harmful, with the potential to influence ecosystems and living organisms. Thorough studies need to be carried out to determine their ecotoxicological effect in order to establish their safe usage in medicine.

### 3.2 Antimicrobial peptides

Antimicrobial peptides (AMPs) are short chains of amino acids that help the innate immune system defend against pathogens. Because of how they work, AMPs are seen as promising tools to fight AMR. Found in all living things, AMPs can target many types of microbes, such as bacteria, fungi, and viruses. They may also have anticancer and immune-modulating effects (Satchanska et al., 2024). AMPs are classified by their structure and function, and they are useful for controlling biofilm growth and related infections in both medical and industrial settings. They can break down biofilms and be used in combination therapies, which make them effective against biofilm-related issues (Fontanot et al., 2024). Natural, semi-synthetic, and synthetic AMPs have all shown effectiveness against biofilms.

Human cathelicidin LL-37 inhibits biofilm development by suppressing quorum-sensing genes, while lactoferrin prevents bacterial adherence (Svensson and Nilsson, 2025). Dendrimeric peptides can prevent and eradicate *Escherichia coli* biofilms, and peptides such as Histatin-5 and defensins impede fungal (Zolin et al., 2021). Defensins have also been reported to prevent significant biofilm formation in *Cryptococcus neoformans* (Andrade et al., 2025). Compared to traditional antibiotics, AMPs offer several advantages: they are less likely to induce resistance, possess a broad target range, and exhibit relatively low toxicity to host cells (Batoni et al., 2011). AMPs are stable, can act synergistically with other medications, and remain effective at reduced concentrations. These characteristics make AMPs promising agents for combating various infections, particularly those associated with biofilms (Table 1).

**Table 1:** The list of some FDA-approved AMPs used to treat bacterial infections.

AMP	Source	Mechanism	Application	References
Bacitracin	*Bacillus subtilis* and *Bacillus licheniformis*	Interference in cell wall and peptidoglycan synthesis		Jin et al. (2021)
Dalbavancin	Semisynthetic lipoglycopeptide	Disruption of cell wall biosynthesis	MRSA infections	Siciliano et al. (2024)
Daptomycin	*Streptomyces roseosporus*	Membrane interruption, inhibition of DNA, RNA and protein synthesis	Skin infections caused by gram positive bacteria	Buttress et al. (2025)
Oritavancin	Semisynthetic glycopeptides	Disruption of gram-positive bacteria cell membrane and inhibition of trans glycosylation and transpeptidation	Complicated skin infections caused by gram positive bacteria	Hussein et al. (2025)
Teicoplanin	Semisynthetic glycopeptides		Gram-positive bacterial infections	Bereczki et al. (2022)
Telavancin	Semi-synthetic derivative of vancomycin	Interference with cell wall and peptidoglycan synthetic	Complicated skin infections caused by gram positive bacteria	Das et al. (2017)
Vancomycin	*Amycolatopsis orientalis*	Inhibition of cell wall synthesis in gram positive bacteria	MRSA infections	Song et al. (2021)
Colistin	*Bacillus polymixa*	Pore formation and disruption of cell membrane	*P. aeruginosa* and other gram-negative bacilli	O’Driscoll et al. (2018)

Despite these advantages, AMPs face substantial challenges in treating biofilm-based infections. Resistance can arise from interactions with EPS within biofilms. Biofilms also activate adaptive resistance responses through sensors such as PhoP/PhoQ and Aps, which induce AMP resistance upon exposure (Singh et al., 2025). The high heterogeneity of bacterial communities within biofilms further complicates treatment by generating diverse susceptibility and resistance profiles to AMPs (Liu et al., 2024). Although AMPs demonstrate broad-spectrum activity and a lower propensity for resistance development compared to conventional antibiotics, their clinical application is limited by significant pharmacokinetic and delivery obstacles (Kim et al., 2025). These peptides are unstable *in vivo* due to rapid proteolytic degradation by host enzymes, including trypsin and chymotrypsin, as well as bacterial proteases (Di, 2015). This instability leads to reduced bioavailability and a short systemic half-life, which is further exacerbated by rapid renal clearance. The cationic nature of AMPs, essential for membrane disruption, is often diminished under physiological conditions with high salt concentrations and serum proteins, thereby reducing antimicrobial efficacy (Oliveira Júnior et al., 2025). At elevated concentrations, AMPs may also induce cytotoxic effects, particularly hemolysis resulting from interactions with erythrocyte membranes, which raises safety concerns. Limited tissue penetration and restricted systemic distribution confine their use primarily to topical applications (Wang et al., 2021).

To address these limitations, advanced strategies such as peptide modification (for example, D-amino acid substitution), nanoencapsulation, and polymer conjugation have been developed to enhance stability, reduce toxicity, and improve targeted delivery, especially for biofilm-associated infections. Furthermore, AMPs possess a narrow safety margin, are rapidly eliminated by the kidneys, and lose significant antimicrobial activity upon contact with biological fluids such as plasma, serum, saliva, and sputum (Oliveira Júnior et al., 2025). These challenges currently hinder the realistic clinical application of AMPs. Ongoing research is investigating various strategies to overcome these barriers. One approach involves identifying peptides that interact minimally with biological fluids, thereby enhancing stability and efficacy. Another strategy employs AMPs in combination with nanoparticles to enhance antibiofilm effects and circumvent certain resistance mechanisms (Raheem and Straus, 2019). Collectively, these approaches aim to improve the efficacy and clinical applicability of AMPs, providing more effective options for the treatment of biofilm-associated infections.

### 3.3 Phage therapy

Bacteriophages are viruses that infect and replicate within bacterial cells, leading to bacterial lysis and death. They vary in size, morphology, and genomic composition, but all contain nucleic acid (DNA or RNA) within a protein capsid, which protects them from environmental stress and aids in host cell delivery. Bacteriophages are typically species-specific, targeting particular bacterial strains (Bisesi et al., 2024). After attaching to a host cell, they replicate via either the lysogenic or lytic cycle. In the lysogenic cycle, the virus replicates without destroying the host cell, while in the lytic cycle, it uses the host’s resources to reproduce, resulting in cell death and the release of new phages. The lytic cycle’s cell-killing and amplification effects form the basis of phage therapy.

Phage therapy is effective against bacterial infections, including those involving biofilms, as bacteriophages do not infect animal cells. Treatment can use a single phage or a combination, with mixtures proving more effective against biofilms. According to Nicola et al., phage therapy offers a narrow range of action, greater safety and tolerability, cost-effectiveness, and targeted specificity with minimal impact on surrounding tissue (Principi et al., 2019). Some successful clinical and experimental studies have further highlighted the rising translational prospective of phage-based therapeutics against MDR infections. Phage therapy has shown potent efficacy in case of chronic infection in cystic fibrosis patients and prosthetic joint infection caused by *Pseudomonas aeruginosa* and *Staphylococcus aureus*, respectively (Peng et al., 2024; Weiner et al., 2025). Both of these cases emphasize the real-world clinical implementation of phage therapy, predominantly in biofilm-associated and antibiotic refractory infections (Ikpe et al., 2024).

Some previous studies indicate that bacteriophages can penetrate the inner structure of biofilms through the water channels present in a biofilm (Lusiak-Szelachowska et al., 2020). Many phages also produce hydrolytic enzymes such as depolymerases and lysins. Depolymerases degrade the extrapolymeric substance (EPS) of biofilms, improving drug permeability and exposing bacteria to antimicrobial agents (Pires et al., 2016). Tait et al. demonstrated that a phage cocktail expressing depolymerase completely eradicated an *Enterobacter cloacae* biofilm (Tait et al., 2002). Lysins hydrolyze the peptidoglycan in bacterial cell walls, causing cell lysis and disrupting biofilms. These properties give phage-delivered lysins both antibacterial and anti-biofilm effects (Lusiak-Szelachowska et al., 2020). Phages lacking these enzymes can be genetically modified to produce them, further enhancing their ability to degrade EPS and inhibit biofilm formation (Hemmati et al., 2021).

**Figure 3:**
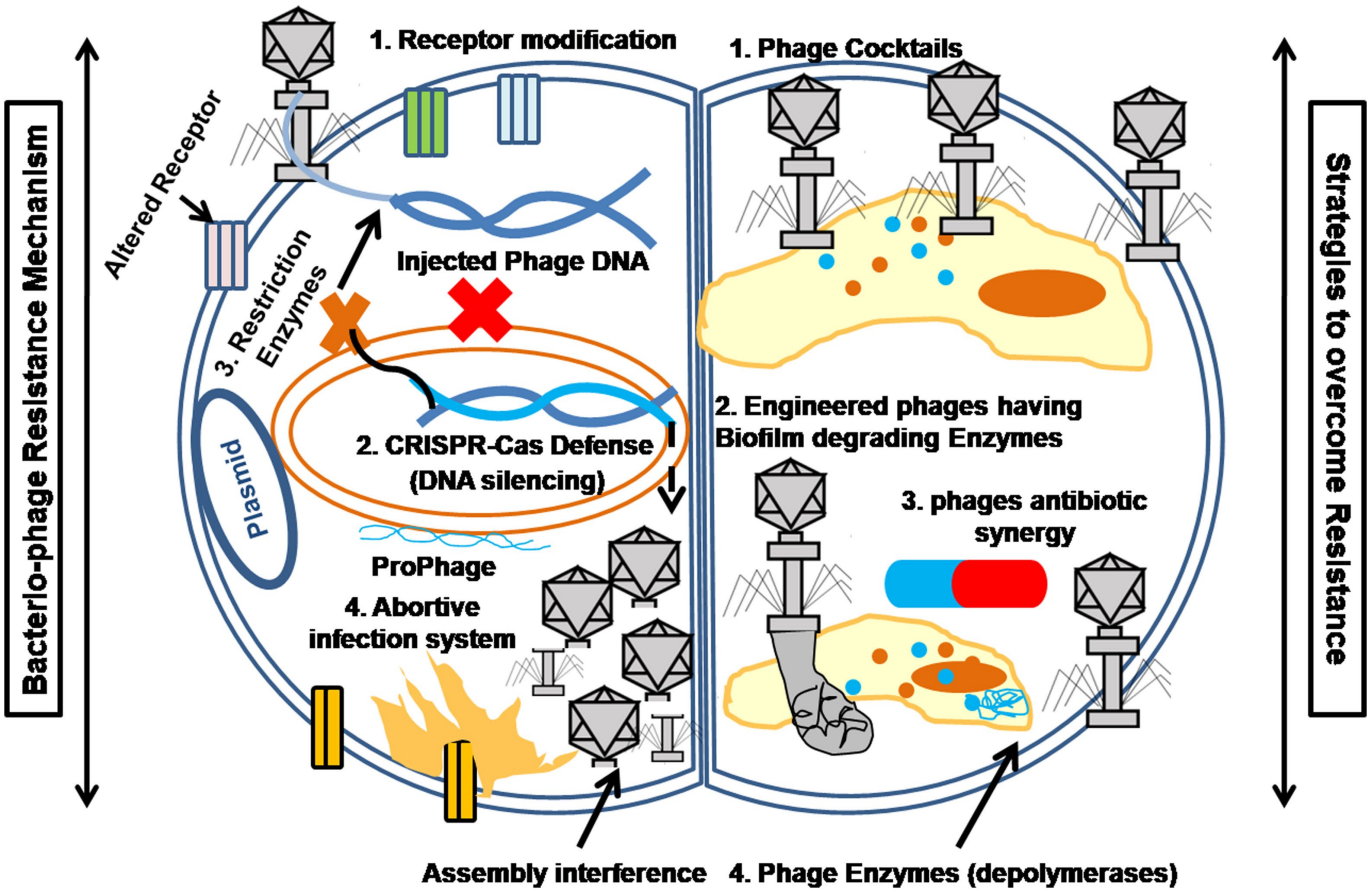
The summarized representation shows the interplay between bacterial defense systems (left panel) and phage-based interventions (right panel).

Although bacteriophages offer significant advantages, bacteria can develop resistance through several mechanisms, as shown in Figure 3. These include altering or losing surface receptors to block phage adsorption, inhibiting phage DNA injection, employing restriction-modification systems to degrade foreign DNA, using CRISPR-Cas adaptive immunity to target phage genomes, and activating abortive infection systems that induce early cell death to limit phage spread (Manley et al., 2024). Together, these mechanisms illustrate the ongoing co-evolution between bacteria and bacteriophages (Shaer-Tamar and Kishony, 2022). In response to such resistance, innovative strategies have emerged. For example, phage cocktails combine multiple phages targeting different bacterial receptors and are widely used to reduce the risk of resistant mutants (Kim et al., 2024). Advances in synthetic biology and genetic engineering have enabled the development of next-generation phages with enhanced capabilities (Alessa et al., 2025). Engineered phages can expand host range, evade bacterial defenses, or deliver CRISPR-Cas systems to selectively eliminate antibiotic resistance genes. They can also be modified to overexpress biofilm-degrading enzymes such as depolymerases and lysins, increasing antibiofilm activity and therapeutic potential (Ferriol-González and Domingo-Calap, 2020; Hemmati et al., 2021).

Beyond these approaches, combination therapy with bacteriophages and antibiotics, known as phage-antibiotic synergy (PAS), has shown promise. Studies indicate this approach enhances bacterial clearance and disrupts biofilms. For example, phage T4 combined with tobramycin significantly reduced tobramycin-resistant *Escherichia coli*. Phage PB-1 was more effective against *Pseudomonas aeruginosa* biofilms (Sahu et al., 2024). Phage–amoxicillin combinations significantly inhibited biofilm formation in *Klebsiella pneumonia* (Gorodnichev et al., 2025).

Despite these successes, challenges remain. Selective pressure may favor antibiotic-resistant strains if phages target only antibiotic-sensitive bacteria. Furthermore, because phage replication relies on bacterial metabolic activity, bacteriostatic antibiotics may hinder phage propagation and complicate treatment outcomes.

Nevertheless, phage-based combination therapies are promising for treating complex and mixed-species infections. In polymicrobial environments, competition among pathogens may further improve therapeutic outcomes (Ghuneim et al., 2022). Optimizing treatment regimens and advancing phage engineering and synthetic biology will be essential to fully realize the potential of bacteriophage-based therapies against antimicrobial resistance and biofilm-associated infections.

### 3.4 Quorum sensing inhibition

Most bacteria use Quorum Sensing (QS) to communicate during biofilm development. QS relies on signaling compounds called Autoinducers (AI) that help bacteria assess their population density. Both gram-positive and gram-negative bacteria produce various types of AI, with some shared systems (Mukherjee and Bassler, 2019). Gram-positive bacteria use Autoinducing oligopeptides (AIP) released from membrane transporters. At sufficient concentrations, AIP bind to histidine kinase sensors to alter gene expression. In *S. aureus,* AIP secretion is regulated by the Accessory Gene Regulator (Agr). Previous reports by Daniel Shin shows that *Pseudomonas aeruginosa* uses N-acyl homoserine lactones (AHL) as signaling molecules in its QS system (Shin et al., 2019). AHL regulates the LuxI/R system, which controls virulence factors and biofilm production. Both bacterial groups also use Autoinducer-2 (AI-2) to communicate with other strains. AI-2 is activated by LuxS synthase, involved in the methyl-cycle and regulating genes related to metabolism, such as those for surface attachment and toxin production (Preda and Sandulescu, 2019).

Studies indicate that disrupting QS pathways can make pathogens more susceptible to the host immune system and antimicrobial agents. Biofilm disruption strategies include quorum quenching, degrading enzymes, and QS inhibitors (QSI). Many QSI molecules are natural plant metabolites, such as cinnamaldehyde, flavonoids, and eugenol. Cinnamaldehyde, a major component of cinnamon, has antimicrobial activity against pathogenic biofilms. Studies show that low concentrations of cinnamaldehyde inhibit QS related to AHL and AI-2 (Preda and Sandulescu, 2019). Cinnamaldehyde can also be combined with other antibiofilm agents, such as colistin and chitosan nanoparticles, to achieve synergistic inhibition and dispersion of *P. aeruginosa* biofilms (Topa et al., 2020; Xu et al., 2022). Other natural compounds, including flavonoids like baicalein and quercetin, have shown QS inhibitory effects, reducing virulence and biofilm formation (Omwenga et al., 2018). Eugenol has been reported to inhibit QS-regulated virulence factors in *Serratia marcescens* (Sethuram et al., 2021).

Evidence suggests that QSIs may be used alongside antibiotics, such as aminoglycosides, β-lactams, tetracyclines, macrolides, quinolones, and polymyxin, to enhance antibacterial activity by disrupting biofilm barriers (Gou et al., 2021). Metal nanoparticles also disrupt biofilms by inhibiting QS signals. Silver nanoparticles (NPs) synthesized from *Piper betle* and other sources suppress QS-mediated virulence in pathogens like *P. aeruginosa and Serratia marcescens* (Srinivasan et al., 2018). Biosynthesized silver NPs are more effective than chemically synthesized ones, although low concentrations may induce QS gene expression. Gold and selenium nanoparticles also block virulence factors and biofilm formation (Elshaer and Shaaban, 2021).

Another approach involves removing signaling molecules using quorum quenching (QQ) bacteria and related enzymes to disrupt cell-to-cell communication (Ziegler et al., 2021). Studies have identified AHL antagonists that degrade AHL-dependent transcription factors, blocking QS pathways. Bacterial isolates that hydrolyze AHL bonds effectively target biofilm-producing *P. aeruginosa* strains. Some marine sediment bacteria and QQ bacterial strains have demonstrated AHL-lactonase activity and effectiveness against MDR pathogens (Khalid et al., 2022). Nanohybrid approaches combining acylase enzymes with metal NPs show increased antibacterial activity. Antibody-targeted QS inhibition using monoclonal antibodies offers potential for treating *P. aeruginosa* infections. Metal-based nanoparticles and novel quorum quenching strategies represent promising methods for inhibiting biofilms and reducing bacterial virulence, particularly in MDR pathogens (Azimzadeh et al., 2025).

Despite the promising potential of QSIs and other anti-biofilm agents, the possibility of resistance development should not be overlooked. Emerging evidence suggests that bacteria may adapt through mutations in quorum-sensing regulatory pathways, modification of signal receptors, or activation of compensatory signaling networks that bypass QS inhibition. In addition, biofilm-associated heterogeneity and stress-response mechanisms may further facilitate adaptive tolerance to these therapies. Therefore, the long-term efficacy of QS-based interventions may depend on their use in combination with conventional antibiotics or other anti-biofilm strategies. Continuous surveillance and antimicrobial stewardship will be essential to minimize the emergence of resistance against this next-generation therapeutics.

### 3.5 CRISPR/Cas system

CRISPR is a groundbreaking technology derived from natural genome editing mechanisms that function as an adaptive immune system in bacteria. It has become a powerful tool in genetic engineering, enabling precise DNA modifications in living organisms and offering therapeutic potential against diseases such as cancer and bacterial infections. The CRISPR system includes a Cas endonuclease and two RNA components, CRISPR RNA (crRNA) and trans-activating CRISPR RNA (tracrRNA) (Adefisoye and Olaniran, 2023). These RNA molecules are often engineered into a single guide RNA (sgRNA), which directs the Cas enzyme to a specific DNA target. Upon binding, the Cas enzyme induces a double-stranded break repaired by error-prone non-homologous end joining, often causing insertions or deletions that generate frameshift mutations and gene knockouts (da Ferreira Silva et al., 2019). CRISPR/Cas systems are classified into two classes and six types based on structural and functional diversity. Class 1 systems (types I, III, and IV) use multi-protein effector complexes, while Class 2 systems (types II, V, and VI) rely on single effector proteins (Janik et al., 2020). Among these, Class 2 Type II CRISPR/Cas9 is the most studied and widely applied in antimicrobial resistance (AMR) research due to its simplicity and ease of use.

Recent studies show CRISPR/Cas systems can target quorum sensing (QS) pathways, which are critical for biofilm formation. Disrupting QS-related genes significantly impairs extracellular polymeric substance (EPS) production and biofilm development. For example, deleting fimH, luxS, and bolA genes in *E. coli* markedly reduced EPS production and biofilm formation (Alshammari et al., 2023). Similarly, plasmid-based CRISPR interference (CRISPRi) targeting the luxS gene inhibited biofilm formation by about 50% in clinical *E. coli* strains (Zuberi et al., 2017). In *Pseudomonas aeruginosa,* downregulating the PA0751 gene reduced bacterial growth, motility, and biofilm formation. Gene silencing of gacS, rimA, bifA, and dipA in *Pseudomonas fluorescens* altered biofilm architecture, including thickness and surface roughness (Noirot-Gros et al., 2019) (Table 2).

**Table 2:** Advantages and limitations of different CRISPR/Cas’s delivery system.

Type of delivery	Delivery method	Delivery format	Advantages	Limitations	References
Viral vectors	Adeno virus, lenti virus, rota virus, bacteriophages	DNA	Can be used in *in vivo*, *ex vivo* and *in vivo* and in studies requiring long-term expression	Low survival rate at infection site Affinity depends on receptors present on bacteria surface	Jia et al. (2023)
	Liposomes and lipid nanoparticles	mRNA and proteins	Can be used in *invivo*, *ex vivo* and *in vivo* with minimal toxicity and immunogenicity	Risk of being encased by endosomes and prevent further delivery processes	Tenchov et al. (2021)
Non-viral vectors	Other nanoparticles	Proteins	High efficiency and lack of immune response and mutagenesis	Low efficacy of action and long-term toxicity	Zhang et al. (2018)
	Conjugative plasmids	ssDNA	Efficient in various CRISPR-Cas9	Depends on donor that needs to be introduced into to replicate	Liang et al. (2017)
	Lopoplexes and polyplexes	Proteins	Less chance of inducing severe immune responses	Less efficiency and transfection efficiencies	Hall et al. (2017)
	Microinjection	DNA; mRNA and proteins	Delivery of any molecular weight of CRISPR component to cytoplasm or nucleus	May induce cell damage and low thought hence not suitable for *in vivo* studies	Wilbie et al. (2019)
Physical vectors	Electroporation	DNA; mRNA and proteins	It can deliver all CRISPR components and is suitable for both *in vitro and ex vivo* studies	Induce cell death and transfection in certain cells and it cannot used for human *in vivo* studies	Fajrial et al. (2020)
	Hydrodynamic injection	DNA; protein	Efficient in various CRISPR-Cas9 and less technically challenging	Not suitable in medical applications for large animals since it can cause trauma, physiological complications and death	Dubey and Mostafavi (2023)

**Figure 4:**
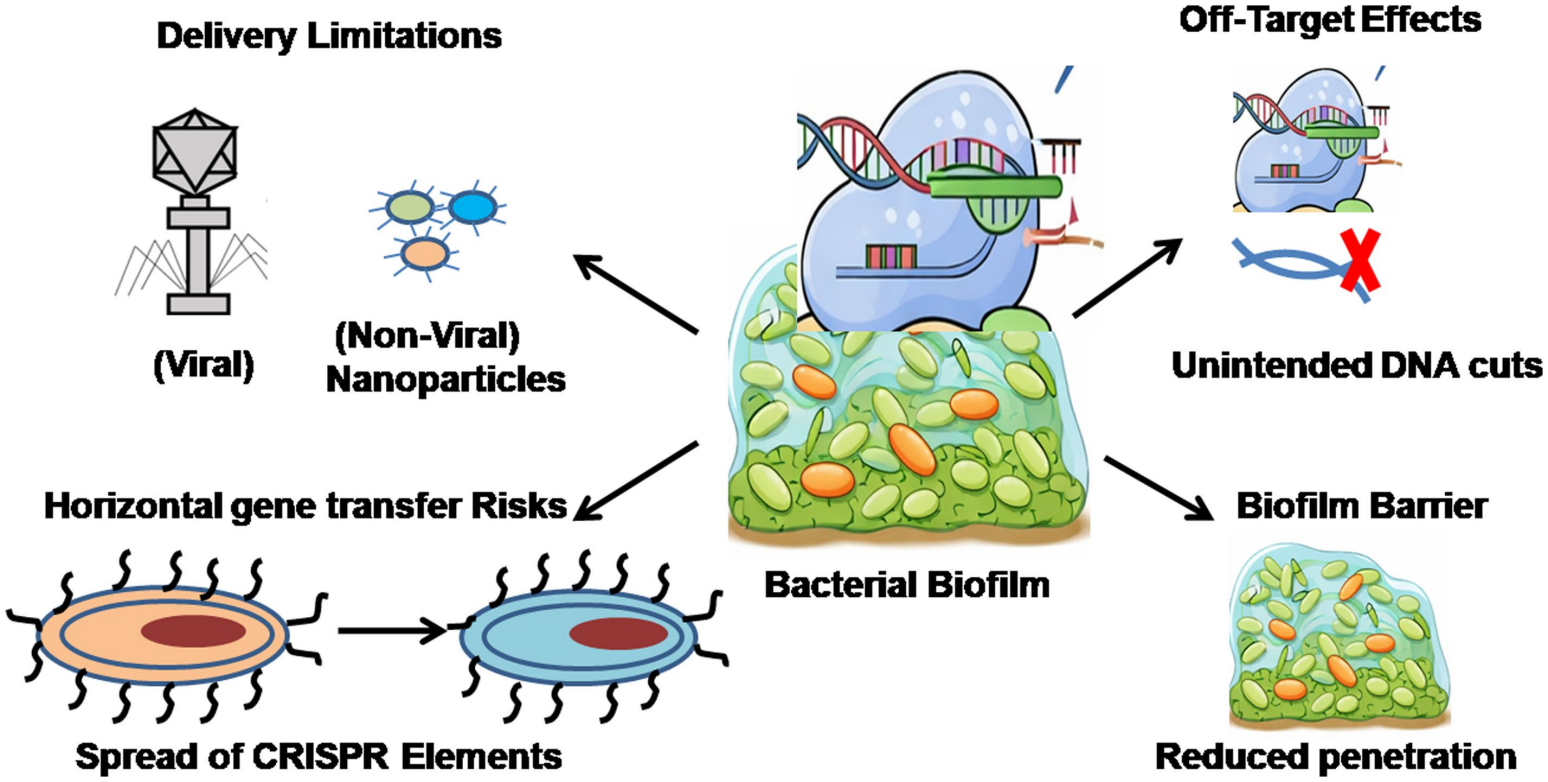
Represents challenges in CRISPR-Cas based antimicrobial therapy.

Although CRISPR/Cas technology is highly specific and versatile, several challenges limit its application as shown in Figure 4. Delivery efficiency remains a primary obstacle, particularly in complex microbial environments such as biofilms, where achieving effective penetration and uniform distribution is difficult. Viral vectors, such as bacteriophages, provide relatively high delivery efficiency but are constrained by a narrow host range, variable bacterial susceptibility, limited stability at target sites, and risks of unintended genetic changes (Fage et al., 2021). Non-viral methods, including physical techniques (electroporation, ultrasonic microbubbles, hydrodynamic injection) and chemical carriers (nanoparticles, liposomes, cell-penetrating peptides), offer alternatives but often result in lower efficiency and may cause cytotoxicity or immune responses (Lee et al., 2025). Conjugative plasmids are also promising, with efficiency similar to that of phages; however, their effectiveness depends on population dynamics, donor strain fitness, and the risk of co-transferring antimicrobial resistance genes (Mayorga-Ramos et al., 2023).

In addition to delivery challenges, off-target effects are a significant concern, as unintended cleavage of non-target genomic regions can disrupt beneficial microbiota or cause unpredictable genetic changes, especially in polymicrobial communities (Guo et al., 2023). The horizontal transfer of CRISPR-associated elements further raises biosafety concerns, as these systems may spread across species, alter microbial ecology, or disseminate engineered traits (Wheatley and MacLean, 2021). While CRISPR/Cas systems show great potential for addressing AMR and biofilm-associated infections, future research should prioritize the development of efficient, targeted delivery systems, reduction of off-target effects, and robust containment strategies to mitigate ecological and evolutionary risks in complex microbiomes.

### 3.6 Strategies based on micro biome engineering and probiotics

To combat the antimicrobial resistance and enhance the host defense mechanism, micro biome engineering and probiotics emerge promising strategies via alteration in composition and functionality of the microbial association. The advance metagenomic studies, and applications of synthetic biology and precision microbiology allows targeted modifications of microbial genomes either by inserting beneficial genes or by modulating consortia to overcome the resistance. Apart from this the probiotics applications enhances the mitigation of AMR by producing antimicrobial peptides, by disruption of horizontal gene transfer, or via degradation of antibiotic entities inside the intestinal gut. The combinatorial approach (combination of both microbiome engineering and next generation probiotics) prevent the emergence of multidrug resistance via sustainable host specific approach to reduce the use of conventional antibiotics (Rabetafika et al., 2023).

### 3.7 Artificial intelligence in combating antimicrobial resistance

Artificial intelligence (AI) is transforming approaches to AMR and biofilm-associated infections. Through data-driven methodologies, AI facilitates the discovery of novel antimicrobial agents, optimizes treatment strategies, and enables the prediction of resistance development. Machine learning (ML), deep learning (DL), and computational modeling empower AI systems to analyze extensive chemical, genetic, and biological datasets. This capability allows researchers to identify potential antimicrobial compounds, inhibitors of bacterial communication, biofilm-disrupting agents, and antimicrobial peptides. These approaches accelerate drug discovery relative to traditional laboratory screening. A significant application of AI in AMR research is drug repurposing, in which existing FDA-approved drugs are evaluated for new antimicrobial or anti-biofilm properties (Mohammed et al., 2025).

Computational tools that integrate virtual screening, molecular docking, and ML-based predictions efficiently examine large chemical libraries for interactions with biofilm-related targets. This strategy reduces time, cost, and regulatory barriers compared to *de novo* drug development. AI also supports the identification of effective drug combinations, optimization of dosing regimens, and personalization of antimicrobial therapies based on pathogen genetic profiles and resistance patterns (Singh, 2024). Increasingly, AI is integrated with high-throughput screening and multi-omics approaches, including genomics, transcriptomics, proteomics, and metabolomics, to identify novel targets and therapeutic strategies for biofilm-associated infections. By analyzing complex datasets from these sources and biofilm experiments, AI can elucidate pathways involved in biofilm development, bacterial communication, and virulence (Sanches et al., 2024).

These insights inform the design of targeted interventions that inhibit biofilm formation, disrupt bacterial signaling, or sensitize resistant bacteria to conventional antibiotics. Predictive modeling tools further assist in monitoring emerging resistance trends and guiding clinical decision-making and antimicrobial stewardship. Recent advancements demonstrate the expanding influence of AI in anti-biofilm research. Supervised ML algorithms such as random forest, support vector machines (SVMs), and gradient boosting have been employed to predict anti-biofilm activity based on molecular and chemical characteristics. For instance, ML models trained on datasets of known biofilm inhibitors have facilitated the identification of small molecules capable of inhibiting biofilm formation in bacteria such as *Pseudomonas aeruginosa*. Deep learning models, including convolutional neural networks (CNNs) and graph neural networks (GNNs), enhance predictive accuracy by capturing complex relationships between molecular structure and biological activity (Mishra et al., 2024).

AI-guided investigations have also yielded novel antimicrobial candidates validated in laboratory settings. Notably, the antibiotic halicin was discovered through deep neural network screening and demonstrated potent activity against multiple drug-resistant bacteria, including *Acinetobacter baumannii*. ML-based approaches have identified synergistic drug combinations that more effectively penetrate biofilms and eradicate bacteria, with several combinations confirmed experimentally (Mulat et al., 2025). Nevertheless, challenges remain in the widespread adoption of AI for anti-biofilm drug discovery. A primary limitation is the scarcity of large, high-quality, and standardized datasets for model training, as biofilm behavior exhibits significant variability across experimental conditions and laboratories. Additionally, many AI-identified compounds demonstrate efficacy only at high concentrations, necessitating the development of more generalizable models and robust experimental validation.

Future progress will rely on integrating microbiological expertise with explainable AI and establishing comprehensive, standardized biofilm datasets to enhance predictive performance. In conclusion, the convergence of AI, conventional drug discovery, and innovative therapeutic platforms presents a promising strategy to address AMR and biofilm-associated infections. Continued advancements in computational modeling, multi-omics integration, and experimental validation are expected to accelerate the development of more precise and effective anti-biofilm therapies (Alanazi, 2023).

## 4 The synergistic approaches to combat AMR

Combinatorial approaches are promising as prevailing strategies to mitigate the biofilm-associated multidrug resistance that are recognized to reduce the effective treatment due to their structural complexity. A synergistic approach, when antibiotics administer in combination with nanoparticles, can enhance the drug penetration and leads to the disruption of the EPS polymeric matrix, meanwhile QSIs combine with β-lactams, reducing the cell signaling and virulence signaling and establishing the antibiotic propensity. Another report suggested that when phage therapy is combined with biofilm matrix-degrading enzymes, it can enhance the matrix distortion and antimicrobial activity to mitigate AMR. The bacteriophages derived enzymes, such as depolymerises and endolysins, have strong efficiency to target and degrade the biofilm extracellular matrix. Therefore, phages containing active forms of these enzymes are efficient to invade extracellular matrix to contribute to enhancing the susceptibility to antimicrobial agents and host immune response (Yahya et al., 2025). Conspicuously, synergistic interactions between bacteriophages and antibiotics have been progressively more examined and reported. The comparative study of combinatorial approaches is shown in Table 3.

**Table 3:** Comparative analysis of combinatorial strategies targeting biofilm-associated multidrug resistance: preclinical and clinical evidence.

Combination strategy	Mechanism of action	Synergistic benefits	Preclinical evidence	Clinical evidence	Limitations/antagonism	References
Nanoparticles + antibiotics	Enhanced drug delivery, EPS penetration, ROS generation	Biofilm penetration and EPS disruption increased antibiotic efficacy	Strong evidence in *in vitro* biofilm studies and animal infection models (e.g., silver, lipid NPs)	Limited; few early-stage translational studies still ongoing	Cytotoxicity, accumulation, regulatory challenges	Afrasiabi and Partoazar (2024)
QSIs + β-lactam antibiotics	Biofilm formation inhibit via Inhibition of quorum sensing pathways	Reduced virulence and disruption of biofilm restored antibiotic susceptibility	Confirmed in *P. aeruginosa* and other pathogens	Mostly preclinical and early translational research	Off-target microbiome effects and incomplete QS inhibition	Rajkhowa et al. (2024)
Bacteriophage + biofilm-degrading enzymes	Depolymerases degrade EPS	Enhanced antimicrobial penetration, targeted killing via biofilm inhibition	*In vitro* and animal model evidence	In translational studies	Enzyme instability, immune neutralization, delivery challenges	Cui et al. (2024)
Bacteriophage + antibiotics	Improved biofilm access via phage and antibiotics mediated cell lysis	Reduced resistance emergence, improved clearance	Strong synergy in biofilm models and infection studies	Confirmed in case-based clinical treatments (e.g., MDR infections)	Possible antagonism if antibiotics inhibit phage replication; immune clearance	Chang et al. (2022)

## 5 Clinical translation barriers

Although anti-biofilm research has advanced considerably, translating these findings into clinically effective therapeutics remains a significant challenge. Numerous biological, practical, and regulatory barriers continue to impede successful development. A primary concern is that *in vitro* anti-biofilm studies often do not accurately replicate the complex microenvironment of chronic human infections. Consequently, therapeutics demonstrating promising efficacy in laboratory settings frequently exhibit diminished effectiveness *in vivo.* Furthermore, several proposed therapeutic strategies present safety concerns, such as off-target effects, cytotoxicity, immune activation, and biocompatibility limitations. Advanced modalities, including bacteriophage therapy, nanoparticle-based delivery systems, AMPs, and CRISPR-based therapeutics, encounter additional obstacles related to consistency, stability, aggregation, degradation, and batch-to-batch variability. These issues complicate regulatory approval processes and large-scale manufacturing.

Clinical variability further increases complexity, as biofilm architecture varies by anatomical location and disease condition, particularly in chronic infections, making the development of universally effective therapeutics challenging. Accelerating clinical translation requires harmonized regulatory frameworks and cost-effective, scalable manufacturing pipelines to ensure reproducibility, safety, and *in vivo* efficacy. Recent technological advancements offer potential solutions. For example, encapsulating bacteriophages within liposomal nanoparticles has enhanced their stability, extended circulation time, and protected them from rapid immune clearance. Genetically engineered phages have shown improved lytic activity and the capacity to deliver biofilm-degrading enzymes directly into the extracellular matrix. Additionally, CRISPR-Cas–encoding phages facilitate sequence-specific targeting of antimicrobial resistance genes within biofilms, representing a highly innovative and precise emerging strategy. Nevertheless, substantial challenges related to regulatory complexity, manufacturing scalability, long-term ecological impact, and biosafety must be addressed before these advanced anti-biofilm therapeutics can achieve widespread clinical adoption.

In addition to biological and technical challenges, regulatory approval pathways, cost-effectiveness, and scalability represent critical barriers to the clinical implementation of advanced anti-biofilm therapies. Innovative approaches such as nanotechnology-based drug delivery systems and CRISPR/Cas-mediated antimicrobials require rigorous evaluation for biosafety, off-target effects, and long-term ecological impact, leading to complex and often prolonged regulatory processes. Furthermore, the high cost associated with development, optimization, and large-scale production of these technologies may limit their accessibility, particularly in low- and middle-income settings. Manufacturing challenges, including reproducibility, stability, and batch-to-batch consistency, further complicates their translation into clinical practice. Addressing these issues will require standardized production protocols, cost-effective design strategies, and harmonized global regulatory frameworks.

## 6 Future strategies

Precision-derived multiple therapeutics may serve as an effective future strategy to combat AMR associated with biofilms. Recent advance techniques such as AI-driven drug discovery, biofilm detection tools, and repurposed drugs will assist clinicians in treating biofilm matrix and resistance determinants. A hybrid strategy, such as biological formations of nanoparticles, AMPs, phages, and CRISPR-based antimicrobials, shows strong potential to penetrate biofilm matrix and is responsible for the disruption of structural and genetic resistance mechanisms. Furthermore, the cocktail of personalized phage–enzyme, engineered probiotics, and synthetic microbial consortia also confers innovative biological alternatives via restoring microbial balance and reducing antibiotic pressure. Parallel, smart materials coated devices used in medical implants and improved *in vivo* models, advanced regulatory frameworks, and scalable manufacturing—will be vital to carry these technologies from stretch to clinical practices. Collectively, all these discussed strategies have the potential to overcome AMR in the clinical settings. For successful clinical translation, equal emphasis must be placed on economic feasibility and large-scale manufacturability alongside technological innovation. The development of cost-effective, scalable, and regulatory-compliant platforms will be essential to ensure that advanced anti-biofilm therapies can be widely implemented in real-world healthcare settings.

## 7 Conclusion

MDR in microbial biofilms remains a major obstacle to effective infection management, particularly in clinical settings. This review highlights several innovative strategies aimed at overcoming biofilm-associated resistance. Advanced materials, including nanoparticles and antimicrobial peptides, have demonstrated promising results in disrupting biofilm architecture and enhancing the efficacy of conventional antibiotics. Novel drug delivery systems, such as liposomes and dendrimers, provide targeted and sustained release, reducing therapeutic dosages and minimizing side effects. Quorum-sensing inhibitors and other anti-biofilm agents offer additional strategies by interfering with microbial communication pathways, thereby preventing biofilm formation and promoting biofilm dispersion. The combination of these approaches with traditional therapies has the potential to improve treatment outcomes significantly. Advancing the fight against biofilm-associated infections requires interdisciplinary collaboration across microbiology, bioinformatics, materials science, and clinical research. Leveraging advanced technologies, including artificial intelligence-driven drug discovery, nanotechnology-based delivery systems, and precision medicine, can improve the specificity and effectiveness of anti-biofilm strategies. Translational validation through robust *in vivo* studies and clinical trials is critical to move laboratory findings into clinical practice. It is also important to consider the risk of microorganisms developing resistance to new anti-biofilm therapies, such as quorum-sensing inhibitors and biofilm-disrupting agents. Adaptive responses and evolutionary pressures within biofilm communities may reduce long-term effectiveness if these strategies are used alone.

Despite their promise, further research is required to elucidate mechanisms of action, optimize formulations, and evaluate safety and efficacy in clinical contexts. Collaborative efforts among researchers, clinicians, and pharmaceutical developers will be critical for translating these innovations from the laboratory to clinical practice. Additionally, implementation should also be accompanied by rigorous stewardship programs to mitigate the emergence of new resistance patterns. Addressing MDR in microbial biofilms necessitates a multifaceted approach that integrates cutting-edge technologies with conventional antimicrobial strategies. The adoption of these novel interventions can advance patient outcomes, reduce healthcare costs, and help combat the global threat of antibiotic resistance.
